# Interactive network analysis of the plasma amino acids profile in a mouse model of hyperglycemia

**DOI:** 10.1186/2193-1801-2-287

**Published:** 2013-06-28

**Authors:** Takayuki Tanaka, Taiga Mochida, Yukihiro Maki, Yasuko Shiraki, Hiroko Mori, Shirou Matsumoto, Kazutaka Shimbo, Toshihiko Ando, Kimitoshi Nakamura, Fumio Endo, Masahiro Okamoto

**Affiliations:** Innovative Science and Technology for Bio-industry, Graduate School of Bioresource and Bioenvironmental Sciences, Kyushu University, 3-1-1 Maidashi, Higashi-ku, Fukuoka, 812-8582 Japan; Department of Pediatrics, Graduate School of Medical Sciences, Kumamoto University, 1-1-1 Honjo, Kumamoto, Kumamoto, 860-8556 Japan; Department of Digital Media, Fukuoka International University, 4-16-1 Gojo, Dazaifu-city, Fukuoka, 818-0193 Japan; Institute for Innovation, Ajinomoto Co., Inc, 1-1 Suzuki-cho, Kawasakiku, Kawasaki, 210-8681 Japan; AminoIndex Department, AJINOMOTO Co., Inc, 15-1, Kyobashi 1-Chome, Chuo-ku, Tokyo, 104-8315 Japan

**Keywords:** Plasma amino acids, Hyperglycemia, Time course data, Interactive network analysis

## Abstract

Amino acids are a group of metabolites that are important substrates for protein synthesis, are important as signaling molecules and play central roles as highly connected metabolic hubs, and therefore, there are many reports that describe disease-specific abnormalities in plasma amino acids profile. However, the causes of progression from a healthy control to a manifestation of the plasma amino acid changes remain obscure. Here, we extended the plasma amino acids profile to relationships that have interactive properties, and found remarkable differences in the longitudinal transition of hyperglycemia as a diabetes emergency. What is especially important is to understand pathogenesis for better treatment and early diagnosis of diabetes. In this study, we performed interactive analysis using time course data of the plasma samples of AKITA mice, which develop hyperglycemia. Primarily, we decided to analyze the interactive property of amino acids which had highly significant association with hyperglycemia, namely alanine, glycine, leucine, isoleucine and valine. Next, we inferred the interactive network structure, which reproduces the actual time course within an error allowance of 10% using an S-system model (a conceptual mathematical model for analyzing and simulating networks). The emphasis of this study was altered interactions of plasma amino acids that show stabilizing and destabilizing features in a variety of clinical settings. By performing sensitivity analysis, the most dominant relations in this network were selected; the control paths from glycine to isoleucine in healthy control and from alanine to glycine in hyperglycemia. This result is in good agreement with the biological knowledge regarding branched-chain amino acids, and suggests the biological importance of the effect from alanine to glycine.

## Background

Observations of metabolic profiles have suggested the possibility of representing biological states and reflect changes in metabolic endogenous homeostasis that are induced by diseases (Raamsdonk et al. 2001; Brindle et al. 2002; Nicholson and Wilson 2003; Allen et al. 2003; An et al. 2004; Sreekumar et al. 2009). This approach is based on the fact that the amounts of the metabolites in biological fluids and tissues change in coordination with the physiological conditions of subjects.

Multivariate analysis and pattern recognition studies have revealed that these metabolic profiles contain phenotypic information that can be used as a signature for a physiological condition. From the viewpoint of metabolic networks in biological systems, amino acids are a group of metabolites that are important substrates for protein synthesis, are important as signaling molecules and play central roles as highly connected metabolic hubs (Felig 1975; Jeong et al. 2000). There are some reports that describe disease-specific abnormalities in plasma amino acid profiles, such as liver failure (Fischer et al. 1975; Soeters and Fischer 1976), obesity and diabetes, which could reflect metabolic modulation induced by insulin resistance (Felig et al. 1969; Felig et al. 1970; Wang et al. 2011). There are some trials that make use of plasma amino acid profiles to diagnose and distinguish abnormal subjects from healthy subjects or subtypes and stages of diseases (Noguchi et al. 2006). One of the traditional examples of using plasma amino acid profiles for diagnostic markers is Fisher’s ratio, which is a ratio of branched-chain amino acids to aromatic amino acids and is used as a diagnostic marker for liver fibrosis (Soeters and Fischer 1976). These studies clearly indicate that the plasma amino acid profile itself can be a useful tool for monitoring the physiological state of an organism.

Although these previous studies discuss how to distinguish different physiological states using the plasma amino acid profile data, the control mechanism behind the change in the profile is not discussed in detail. Therefore, conventional investigations have room for improvement at determining why the plasma amino acid profiles change in accordance with the physiological state and which amino acid is the trigger to shift from a healthy control to a manifestation.

To solve this problem and understand the interactive mechanism that controls the plasma amino acid concentrations under physiological conditions, an analysis of the interactive properties of the network is necessary.

In the interactive modeling approach, various network analysis models are proposed. The advantage of the understanding of the interaction between these system components by network analysis is to be able to infer the interrelated and directional network structure of the system components without considering any prior topological information. This analysis should lead us to further understand the interaction of the profiles of system components and provide some clues for the factors that affect the interrelated network under various physiological conditions. Using the time courses of the data, the interactive network structure was investigated using the S-system analysis method (Savageau 1976; Maki et al. 2004; Shikata et al. 2010; Somogyi and Sniegoski 1996; Savageau 1998; Maki et al. 2001; Kikuchi et al. 2003). The S-system is one of the best formalisms for estimating the interactive mechanisms among the system components and enables us to reconstruct the network architectures with the experimentally observed time courses of the quantities of the network components. Its greatest appeal is its simpler two-term format, which leads to unusual mathematical opportunities, which we will discuss later. In particular, the steady state of an S-system can be computed with methods of linear algebra, whereas other formats require more complicated methods (Mueller et al. 1998; Savageau 1993). Maybe surprisingly, the S-system format is also the more accurate representation for functions with a hyperbolic shape, that is, functions that start at a small value and monotonically grow toward saturation, such as a Michaelis-Menten rate function.

In this study, we performed interactive network analysis while using time course data of the plasma samples of AKITA mice, which develop hyperglycemia, and the analytical method based on the S-system model. Furthermore, we performed sensitivity analysis for each interrelated control path in the network model and determined which paths or relations between amino acids are dominant in maintaining the network structure.

## Results

### Time course data of the experimental measurements and calculations in Ins2+/+ and Ins2+/−

**Figure 1:**
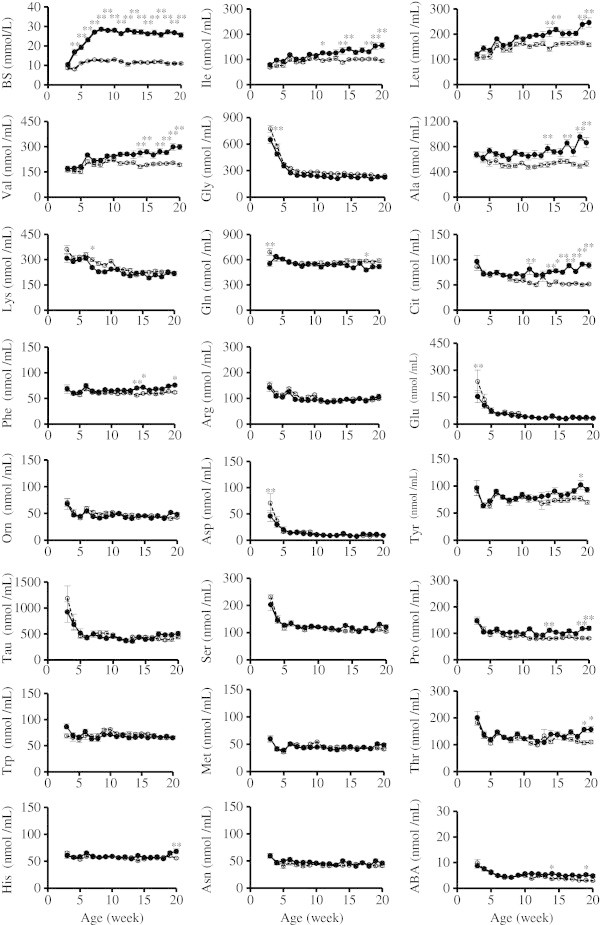
**The changes in BS and plasma amino acids in Ins2+/+ and Ins2+/−.** Line graph for the change in the fasting BS and representative plasma amino acids from 3 to 20 weeks of age. To compare the change in the fasting BS and plasma amino acids between the Ins2+/− and Ins2+/+ mice, statistical analysis was performed using two-way ANOVA, and Bonferroni correction was used for the post hoc test. Data are the mean ± standard error of the mean. The solid line indicates Ins2+/− (n = 9–13). The dotted line indicates Ins2+/+ (n = 8–12). * *p* < 0.05, ** *p* < 0.01, Ins2+/− vs. Ins2+/+. This figure was revised based on our previous study (Mochida et al. 2011).

**Figure 2:**
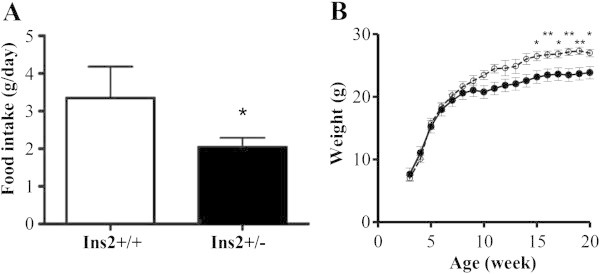
**Food intake and the changes in weight in Ins2+/+ and Ins2+/−. A**. Bar graph for the food intake in Ins2+/− and Ins2+/+. The vertical axis indicates the food intake (g/day). The horizontal axis indicates the age (week). Data are the mean ± SEM. The white bar indicates Ins2+/+ (n = 3). The black bar indicates Ins2+/− (n = 3). Food intake was lower in Ins2+/−. *p < 0.05, Ins2+/− vs. Ins2+/+. **B**. Line graph for the change in weight from 3 to 20 weeks of age. The vertical axis indicates the body weight (g). The horizontal axis indicates the age (week). Data are means ± SEM. The solid line indicates Ins2+/− (n = 9–13). The dotted line indicates Ins2+/+. (n = 12). * *p* < 0.05, ** *p* < 0.01, Ins2+/− vs. Ins2+/+. This figure was revised based on our previous study (Mochida et al. 2011).

The time courses of the blood sugar (BS) and the plasma amino acids are shown in Figure 1. At 3 weeks of age, BS was not different between a mouse model of hyperglycemia (Ins2+/−) and a mouse model of healthy control (Ins2+/+). The Ins2+/− BS had started to become elevated by 4 weeks of age. It continued to elevate until about 7 weeks of age. After that, there was little BS change in Ins2+/−. On the other hand, the Ins2+/+ BS hardly changed until 20 weeks of age. As compared with Ins2+/+, BS was significantly higher in the Ins2+/− mice after 4 weeks of age (Figure 1). In comparison with Ins2+/+, the plasma concentrations of isoleucine, leucine, valine, alanine, citrulline, phenylalanine, proline and histidine were significantly higher and those of glycine, glutamine, glutamate and asparagine were significantly lower in Ins2+/− mice (P < 0.01, age-by-genotype interaction). In association with continuous hyperglycemia, isoleucine, leucine, valine, alanine and glycine showed top-ranked correlations with BS in plasma amino acids (Table 1). As shown in Figure 1 and Table 1, isoleucine, leucine, valine, alanine and glycine showed the significant difference of the longitudinal change between Ins2+/+ and Ins2+/− and top-ranked correlation with BS in plasma amino acids, which indicated that the longitudinal changes in these plasma amino acids had highly significant association with hyperglycemia. In comparison with Ins2+/+, food intake was lower in Ins2+/− at 12 weeks of age (P < 0.05). Weight was significantly lower in Ins2+/− mice after 15 weeks age (P < 0.05, age-by-genotype interaction) (Figure 2).

**Table 1:** **Pearson’s correlation coefficients between the BS and the plasma amino acids**

	Coefficient of correlation with BS	
	r	***p***value
Ile	0.50	2.15E-21
Leu	0.50	2.15E-21
Val	0.54	2.15E-21
Gly	−0.43	1.55E-20
Ala	0.36	7.14E-14
Lys	−0.31	1.63E-10
Gln	−0.28	6.92E-09
Cit	0.26	6.36E-08
Phe	0.25	1.70E-07
Arg	−0.24	6.38E-07
Glu	−0.21	1.26E-05
Orn	−0.20	2.85E-05
Asp	−0.20	4.57E-05
Tyr	0.20	6.03E-05
Tau	−0.16	1.05E-03
Ser	−0.14	4.40E-03
Pro	0.13	1.04E-02
Trp	−0.11	2.56E-02
Met	−0.06	2.00E-01
Thr	0.05	2.82E-01
His	0.03	4.92E-01
Asn	0.02	6.14E-01
ABA	−0.01	8.89E-01

Pearson’s correlation coefficients were used to evaluate the correlations between the BS and the plasma amino acids of the time-courses.

**Figure 3:**
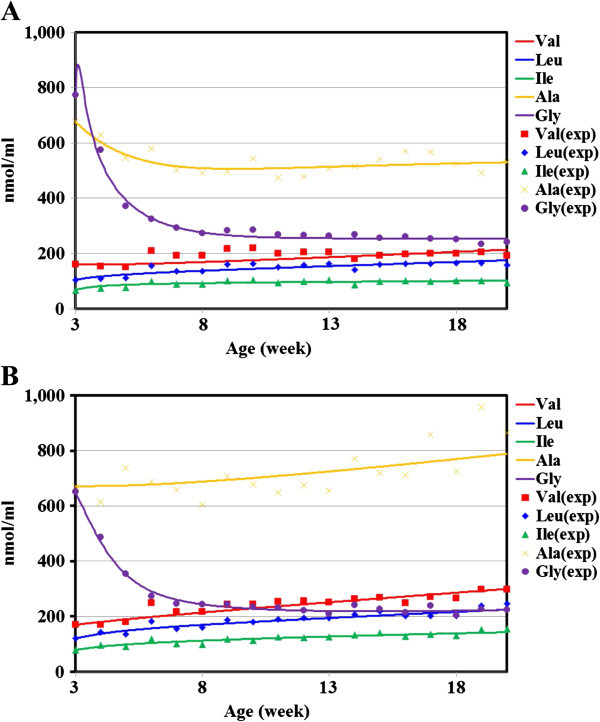
**Plasma amino acid time-course calculated using the parameter set in Table**2**.** The experimentally measured concentrations of each amino acids from 3 to 20 weeks of age are indicated with symbols and the calculated bold lines were obtained with the estimated parameters shown in Table 2. **A**. Time-course data of the experimentally measured and that of the calculated in Ins2+/+. **B**. Time-course data of the experimentally measured and that of the calculated in Ins2+/−.

In Ins2+/+ (Figure 3A), the concentration of alanine, leucine, isoleucine and valine slightly fluctuated throughout the experimental period. A decrease in the concentration of glycine was observed in week 3. By week 9, this concentration was decreased by approximately one-third of the concentration measured before the experiment. In Ins2+/− (Figure 3B), the concentration of alanine, leucine, isoleucine and valine fluctuated slightly and gradually increased more than those of the Ins2+/+ throughout the experimental period. A decrease in the concentration of glycine was observed in week 3. By week 9, this concentration was decreased by approximately half of the concentration measured before the experiment.

### Optimization of the parameter set

Using the time courses of the data described above, the interactive network structure was investigated using the S-system analysis method (Savageau 1976). The S-system is one of the best formalisms for estimating the interaction mechanisms among the system components and enables us to reconstruct the network architectures with the experimentally observed time courses of the quantities of the network components. The inference of the interactive network structure is achieved by optimizing the S-system parameter set. For the evaluation of the network structure defined by each parameter set, the average relative error between the calculated value and experimental value per sampling point was computed, and the network model that gave the least average error was evaluated highly.

**Figure 4:**
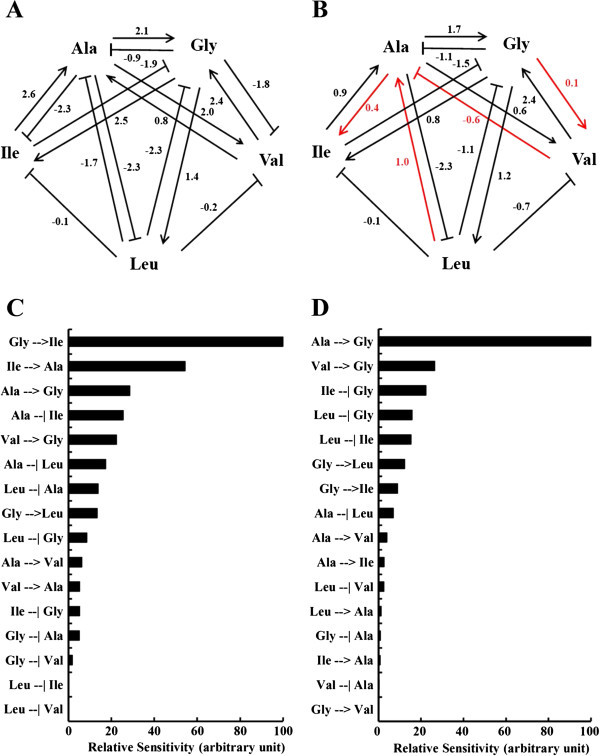
**The network model defined by the parameter set in Table**2**.** The arrows indicate positive effects, and the lines with bars indicate negative effects. The numbers adjacent to the lines indicate the interrelated coefficient of each path. Red arrows and lines with bars indicate the difference between Ins2+/+ and Ins2+/−. **A**. Ins2+/+ network model defined by the parameter set in Table 2. **B**. Ins2+/− network model defined by the parameter set in Table 2. Relative sensitivity of each of the parameters in the network model. The arrows indicate positive effects from amino acids on the left to amino acids on the right, and the bars indicate negative effects from amino acids on the left to amino acids on the right. **C**. Ins2+/+ relative sensitivity of each of the parameters in the network model. **D**. Ins2+/− relative sensitivity of each of the parameters in the network model.

After 50 trials of optimization by the genetic algorithm described in the Methods section, one network model was selected in each group; this network model had a structure that reproduced the experimentally obtained time courses with the smallest average relative error. The obtained parameter set of this model is shown in Table 2; there, the interrelated coefficient of each path in the network is indicated as a matrix, in which the amino acid giving the influence (*j*) is indicated in the top row and the amino acid (*i*) receiving the effect is indicated in the left column. For each network component which is an amino acid (*X*_*i*_), the non-negative parameters *α*_*i*_ and *β*_*i*_ determine relative inflow and outflow of *X*_*i*_. The terms *g*_*ij*_ determine the interactive affectivity of amino acid *X*_*j*_ to amino acid *X*_*i*_*g*_*ij*_ represents the interrelated coefficient of *X*_*j*_ to the synthesis of *X*_*i*_. The terms *h*_*ii*_ determine the interactive affectivity of amino acid *X*_*i*_ to amino acid *X*_*i*_; *h*_*ii*_ represents the interrelated coefficient of *X*_*i*_ to the degradation of *X*_*i*_. In this case, the value of other *h*_*ii*_ (i ≠ j) was assumed to be 0. The number of estimated parameters in this S-system formalism is *n* × (*n* + 3), where *n* is the number of state variables (*X*_*i*_). This network model reproduced the actual time course with an average error of 7.37% in Ins2+/+ and 6.63% in t Ins2+/− (Figure 3A and B). The same network model is visualized as a network figure in Figure 4A and B. In the interactive network (Figure 4A and B), sign reversal of the interrelated coefficient was observed in valine to alanine, leucine to alanine, isoleucine to alanine, alanine to isoleucine and glycine to valine between Ins2+/+ and Ins2+/−. However, the dynamic range of the interrelated coefficient was maintained not only with the common network structure of Ins2+/+ and Ins2+/− but also with the network structure of Ins2+/+ in a direction opposite to Ins2+/−.

**Table 2:** **The parameter set defining the interactive network model**

(Ins2+/+, Ins2+/−)		***g***_***ij***_						***h***_***ij***_				
		***j***						***j***				
***i***	***α***	Val	Leu	Ile	Ala	Gly	***β***	Val	Leu	Ile	Ala	Gly
Val	(1.5, 4.4)	(0, 0)	(−0.2, -0.7)	(0, 0)	(2.0, 0.6)	(−1.8, 0.1)	(1.0, 1.5)	(0, 0.01)	(0, 0)	(0, 0)	(0, 0)	(0, 0)
Leu	(7.5, 4.8)	(0, 0)	(0, 0)	(0, 0)	(−1.3, -1.0)	(1.4, 1.2)	(1.2, 1.0)	(0, 0)	(0, 0)	(0, 0)	(0, 0)	(0, 0)
Ile	(7.6, 3.8)	(0, 0)	(−0.1, -1.3)	(0, 0)	(−2.3, 0.4)	(2.5, 0.8)	(1.1,1.5)	(0, 0)	(0, 0)	(0, 0)	(0, 0)	(0, 0)
Ala	(7.4, 4.6)	(0.8, -0.6)	(−1.7, 1.0)	(2.6, 0.9)	(0, 0)	(−0.9, -1.1)	(1.2, 1.5)	(0, 0)	(0, 0)	(0, 0)	(0.7,0)	(0, 0)
Gly	(8.8, 2.8)	(2.4, 1.2)	(−2.3, -1.1)	(−1.9, -1.5)	(2.1, 1.7)	(0, 0)	(1.2, 5.6)	(0, 0)	(0, 0)	(0, 0)	(0, 0)	(1.3, 0.8)

The interrelated coefficient of each path in the network model (left: Ins2+/+, right: Ins2+/−) is indicated as a matrix in which the amino acid giving the influence (j) is indicated in the top row and the amino acid receiving the effect (i) is indicated in the left column. The variable g_ij_ indicates the effect between each pair of amino acids, and h_ij_ indicates the speed of degradation for each amino acid. The parameters with value 0 indicate that there is no relationship between the two amino acids.

### Sensitivity analysis

For the parameter set of the network model shown in Table 2, we performed sensitivity analysis. The sensitivity of each parameter is evaluated by adding a 5% error to the estimated value of the parameter and quantifying the difference that is generated over the time course. Larger differences more strictly regulate the parameter; thus, the parameters that cause large differences are dominant in determining the network structure. For convenience of comparison, the sensitivity of each parameter is scaled to relative values, with the highest value being 100.

The relative sensitivity of each parameter in the network model is shown in Figure 4C and D. The most dominant interactions were the positive effect of glycine on isoleucine in the Ins2+/+ (Figure 4C) and positive effect of alanine on glycine in Ins2+/− (Figure 4D). As shown in Figure 4C and D, the value of the relative sensitivity and differences between the top-ranked and other sensitive parameters of the Ins2+/+ are smaller than those of Ins2+/−. Compared with the Ins2+/+ in Figure 4C, the rank of the positive effect of glycine to isoleucine in Ins2+/− dropped drastically and that of alanine to glycine increased with the relative sensitivity, as shown in Figure 4D.

## Discussion

Metabolism can be viewed as a network that can become perturbed during disease and physiologic insults. In addition to the metabolic pathway map within cells, there are various levels of networks within the body, e.g., the transport of substrates such as amino acids in the blood, moving between organ systems, which represents one level.

With the time course data of the plasma amino acid concentrations obtained under Ins2+/+ and Ins2+/−, an interactive network model was inferred in the form of an S-system differential equation model. The network model could reproduce the experimentally obtained time courses of the plasma amino acids with an average error of 7.37% in Ins2+/+ and 6.63% in the Ins2+/− (Figure 3). The size of the analytical experimental error estimated with the coefficient of variation (Cv) was 10.07% in this experiment. Because the average error for the network model is smaller than the analytical error, we presumed that the model reproduced the experimental time course within the permissible error range.

The concentration of isoleucine, leucine, valine, alanine and glycine was shown to be different in terms of the longitudinal changes between Ins2+/+ and Ins2+/− and showed top-ranked correlation with BS in plasma amino acids (Figure 1 and Table 1). The biological mechanism underlying these metabolic changes in amino acid profiles can be partly understood based on metabolic shifts resulting from a catabolic shift in protein metabolism and upregulated gluconeogenesis (Chevalier et al. 2006), partly because food intake and weight of Ins2+/− were lower than those of Ins2+/+ (Figure 2). After 15 weeks of age, that is to say after the onset of hyperglycemia, weight was significantly lower in the Ins2+/− as compared with Ins2+/+. Moreover, the plasma insulin of Ins2+/− tends to be lower than that of Ins2+/+ (Mochida et al. 2011). Altered plasma amino acid levels could be explained by enhanced protein catabolism associated with the lower insulin level. Although it would be necessary to measure a catabolism-related marker. The metabolic state of hyperglycemia is closely related to the pathophysiological state of metabolic syndrome associated with diabetes (Després and Lemieux 2006). In a recent study on metabolic profiles predicting diabetes, several amino acids, including branched-chain, showed significant associations with future occurrences of diabetes (Wang et al. 2011). It is interesting to point out the close relationship between this study in human and ours in AKITA mice and the importance of amino acid profiles in predicting future diabetes and diagnosing existing hyperglycemia, although the study design and target were different.

Our results showed significant differences in plasma amino acids in accordance with the degree of hyperglycemia. We found that there were higher levels of branched-chain amino acids and alanine in Ins2+/−, while there were lower levels of glycine. These observations have some relation with the sign reversal of the interrelated coefficient in valine to alanine, leucine to alanine, isoleucine to alanine, alanine to isoleucine and glycine to valine between Ins2+/+ and Ins2+/− (Figure 4A and B). Metabolic alterations, such as the sign reversal of the interrelated coefficient, could contribute to altered amino acid levels in response to hyperglycemia.

From the sensitivity analysis, the parameters in the network were ranked by their dominance in determining the network structure. As shown in Figure 4C and D, the most dominant interaction was the positive effect of glycine to isoleucine in Ins2+/+ and positive effect of alanine to glycine in Ins2+/−. In the values for sensitivity, the difference between the top-ranked and other sensitive parameters of Ins2+/+ is smaller than that of Ins2+/−. What is notable is that the sensitivity of Ins2+/− is concentrated in the positive effect of alanine to glycine. Therefore, it is expected that Ins2+/− regulates the sensitivity of the kinetic parameters more intensively than Ins2+/+, and certain relations between amino acid concentrations are specific for certain physiological phenomena; thus, individuals whose physiologic states are different could have a different pattern in the network structure that follows (Noguchi et al. 2006).

The positive effect of glycine to isoleucine was the most dominant interaction in the network model for Ins2+/+ (Figure 4C). There are some observations with this relationship. It has been reported that increased branched-chain amino acids, including isoleucine, could contribute to increased gluconeogenesis and glucose intolerance via the transamination of pyruvate to alanine (Newgard et al. 2009). In addition, increases in alanine, a highly gluconeogenic amino acid, activate the glycine–pyruvate metabolic linkage. Two of the most dominant interactions that had a relative sensitivity over 50% in the Ins2+/+ had a positive effect of glycine to isoleucine and positive effect of isoleucine to alanine (Figure 4C). Compared with the Ins2+/+ in Figure 4C, the rank of the positive effect of glycine to isoleucine and that of isoleucine to alanine in Ins2+/− dropped drastically with the relative sensitivity, as shown in Figure 4D. The sensitivity of the target kinetic parameter in Figure 4C and D can be evaluated by the total amount of difference between the experimentally measured time courses of the target reactants and the calculated values for the case of % change in the value of the target kinetic parameter. The higher the sensitivity, the more strictly regulated the parameter; thus, the parameters that have a high sensitivity are dominant in determining the network structure.

Thus, as shown in Figure 4C, the first and second top-ranked sensitive parameters in the Ins2+/+ (the positive effect of glycine to isoleucine and isoleucine to alanine, respectively) are assumed to play a role in preventing hyperglycemia; however, as shown in Figure 4D, the sensitivity of these two parameters has drastically decreased in ranking, and the positive effect of alanine to glycine has alternatively increased in ranking. Therefore, it is expected that this switch in sensitivity is the trigger to the shift from the healthy control to hyperglycemia.

The positive effect of alanine to glycine in Ins2+/− can be explained by upregulated gluconeogenesis (Figure 4D). In terms of metabolic mechanisms, glycine and alanine are typical glucogenic amino acids. Relevant metabolic pathways generate glucogenic metabolites in the tricarboxylic-acid cycle and also via the glycine–pyruvate metabolic linkage and alanine shuttle in gluconeogenesis. Alanine is known to be a major gluconeogenic precursor. It has been reported that alanine increased in Ins2+/−, and the BS itself was the most important factor related to the alanine metabolism rather than insulin secretion (Robert et al. 1985; Shulman et al. 1980). It has been reported that in hepatocytes, glucose production from glycine increases in diabetic individuals, while this type of glucose production is low under healthy conditions (Hetenyi et al. 1988). Glycine is synthesized from glycolytic intermediates via 3-phosphoglycerate dehydrogenase, which is an NAD-linked enzyme that converts 3-phosphoglycerate to 3-phosphohydroxypyruvate (Noguchi et al. 2008).

Thus, changes in glyceroneogenesis because of an increased demand for glycerol and glyceride for triglyceride synthesis can affect glycine levels in the peripheral circulation.

When the concentration of alanine increases, the demand for glycerol and glyceride for triglyceride synthesis also increases, which accelerates glyceroneogenesis and activates the glycine–pyruvate metabolic linkage. Thus, the concentration of alanine has a positive effect on concentrations of glycine. In view of the positive effect of alanine to glycine in Ins2+/− compared with the Ins2+/+, as shown in Figure 3A, it is expected that the concentration of alanine and glycine increase with Ins2+/−, as shown in Figure 3B. In contrast to expectation, the concentration of glycine decreases. However, the concentration of alanine increases as expected. In Ins2+/−, it is assumed that the supply of glycine and alanine is upregulated, and the utilization of glycine is much more upregulated than its supply. This result demonstrates the advantage of applying network analysis to plasma amino acid data when determining the interactive mechanism, such as the balance between the supply and utilization of plasma amino acids in accordance with the physiological state.

Metabolic changes in amino acids can be better understood as a catabolic shift in protein metabolism and upregulated gluconeogenesis that is induced by the insulin secretory response. Although our analysis did not consider any topological information on amino acid metabolism and was completely data-driven, interactions determined to be dominant by systems analysis and sensitivity analysis are well-known interactive relations in glyceroneogenesis. This fact indicates that our analytical methods are useful in finding the important relations between amino acids with and without any prior knowledge of the amino acid metabolism. It also indicates that the positive effect of glycine to isoleucine and that of alanine to glycine, which were selected with the same analytical procedure, might have some physiologically important function.

## Conclusions

We have shown an attempt to explore interactive mechanisms of plasma amino acids subjected to physiological properties of the longitudinal transition in a mouse model of hyperglycemia. On the basis of these approaches, it might be able to show that, in the combination with experimental data, computational biology has clear added value and that on the basis of the findings a number of expected results can be extracted from the network analyses as well. In this study, we demonstrated the advantage of applying network analysis to plasma amino acid data for constructing a hypothesis about why the plasma amino acid profiles change in accordance with physiological states.

## Methods

### Animals, experimental design and sample collection

We used male mice that were obtained by mating male and female heterozygous C57BL/6-^Ins2 AKITA^/J mice purchased from the Jackson Laboratory in Bar Harbor, Maine, USA. Experimental procedures for the treatment of the mice were approved by the Center for Animal Resources and Development (CARD) of Kumamoto University. Animal care was performed as outlined in the Guide for the Care and Use of Laboratory Animals. The AKITA mouse has a missense mutation of the insulin 2 gene (Ins2). This mutation is inherited in an autosomal dominant manner. Homozygous male mice (Ins2−/−) develop hyperglycemia by 2 weeks of age (Kayo and Koizumi 1998). All Ins2−/− mice displayed hyperglycemia at 3 weeks of age. Ins2−/− mice were excluded from this study because their plasma amino acids could not be determined before the increase in BS. The wild type male mice (Ins2+/+) (n = 8–12) do not develop hyperglycemia (Kayo and Koizumi 1998). Heterozygous male mice (Ins2+/−) (n = 9–13) develop hyperglycemia between 4 and 8 weeks of age (Oyadomari et al. 2002). The development of hyperglycemia is not associated with either obesity or insulitis but is associated with pancreatic β-cell dysfunction (Wang et al. 1999). Diabetic complications manifest in the Ins2+/− mice until 20 weeks of age (Haseyama et al. 2002; Hong et al. 2007; Schmidt et al. 2009).

Starting from 3 weeks of age, mice were maintained on a 12:12-h light–dark cycle, with water provided freely. The composition of the diet was based on a CE-2 (Clea Japan, Inc., Tokyo, Japan) standard diet under ad libitum feeding conditions. Between 3 to 20 weeks of age, blood samples were collected from the tail vein. After the body weight measurement, we performed blood sampling from 11:00 to 12:00 after fasting for 2 hours once every week, and we measured the BS and plasma amino acids in the mice.

For the blood sampling, the mice were anesthetized with 3% isoflurane. The blood samples were collected with capillaries (Ringcaps; Hirschmann Laborgeraete GmbH & Co. KG., Germany) containing EDTA-2Na and kept in a tube at 4°C. The amount of blood was 100 μL for the mice that were more than 5 weeks of age and 75 μL for the mice that were less than 4 weeks of age. Fasting BS was determined by the immobilized enzyme electrode method (Glutest Ace R; Arkray Factory, Inc., Japan). For the extraction of the plasma sample, the collected blood was centrifuged at 1500 × *g* for 10 minutes at 4°C. The plasma samples were kept at −80°C until the plasma amino acids were measured. The amino acids were measured using high-performance liquid chromatography-electrospray ionization mass spectrometry (Shimbo et al. [Bibr CR35][Bibr CR36]).

### Statistical analyses

Variables were expressed as mean ± standard error of the mean. To compare the longitudinal change, statistical analysis was performed using two-way ANOVA, and Bonferroni correction was used for the post hoc test. To evaluate the correlations between the BS and plasma amino acids, pearson’s correlation coefficients were calculated. To compare the food intake between Ins2+/+ and Ins2+/−, statistical analysis was performed using Student's *t*-test.

### Interactive network analysis

We analyzed the interactive properties of amino acids which had highly significant association with hyperglycemia showing the significant difference of the longitudinal change between Ins2+/+ and Ins2+/− and top-ranked correlation with BS in plasma amino acids.

In the interactive network analysis, the interrelated coefficients between the amino acids were first determined, and then, each path was subjected to sensitivity analysis for its dominance in maintaining the network structure.

### S-system analysis method

The S-system belongs to the type of power-law formalism in which the component processes are expressed by differential equations (Savageau 1976):1

where *n* is the total number of state variables or network components (*X*_*i*_), and *i* and *j* (1 ≤ *i*, *j* ≤ *n*) are suffixes of state variables. The terms *g*_*ij*_ and *h*_*ij*_ are the interactive affectivity of *X*_*j*_ to *X*_*i*_; *g*_*ij*_ (*h*_*ij*_), which represents the interrelated coefficient of *X*_*j*_ to the synthesis (degradation) of *X*_*i*_. Thus, the first term of the equation represents all of the influences that increase *X*_*i*_, whereas the second term represents all of the influences that decrease *X*_*i*_. In a network context, the non-negative parameters *α*_*i*_ and *β*_*i*_ are called the relative inflow and outflow, respectively, of network component *X*_*i*_, and real-valued exponents *g*_*ij*_ and *h*_*ij*_ are referred to as the interrelated coefficients between components *X*_*j*_ and *X*_*i*_, respectively.

To infer the network model, the parameters *α*_*i*_*, β*_*i*_*, g*_*ij*_ and *h*_*ij*_ must be optimized. In this optimization problem, each set of parameter values to be estimated is evaluated using the following procedure: suppose that  is the numerically calculated time course of the state variable *X*_*i*_ at time *t* and that  represents the experimentally observed time-course of *X*_*i*_ at time *t*. We sum the relative error in *X*_*i,cal,t*_ to obtain the total error *E*2

where *N* is the total number of experimentally observable state variables, and *T* is the total number of sampling points over time in one experimental condition. The computational task is to determine a set of parameter values that minimizes the objective function *E* within a given error allowance.

**Figure 5:**
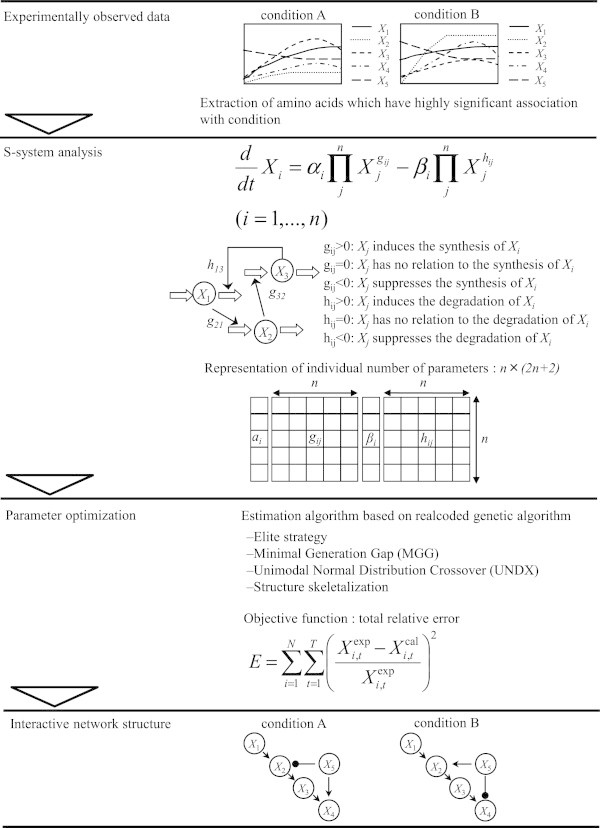
**The scheme of interactive network analysis using the S-system.** First, the time course data were obtained, and the S-systems analysis was performed, calculating the time-courses for each parameter set using a real-coded genetic algorithm and evaluating the average relative error from the experimental data. Finally, the inferred network model was verified for its applicability to simulating other time courses.

For the optimization of a large number of real-valued parameters, the computational technique based on real-coded genetic algorithms (RCGA) (Nakatsui et al. 2008; Ono and Kobayashi 1997; Ueda et al. 2002) is introduced as a nonlinear numerical optimization method that is much less likely to become stuck in a local minimum. This technique is based on a combination of UNDX (Ono and Kobayashi 1997) and an alteration of the MGG model (Sato et al. 1997). The genome (design code) of each individual (each set of parameter values) is shown in Figure 5. A genome (corresponding to one individual) contains a set of S-system parameters (*n α*_*i*_ s and *β*_*i*_ s and *n* × *n g*_*ij*_ s and *h*_*ij*_ s), which form an *n* × (2*n* + 2) matrix. An individual represents one S-system model. Each small square in Figure 5 corresponds with each parameter that has a real value.

### Parameter optimization

The interactive analysis was performed using amino acids which had highly significant association with hyperglycemia showing the significant difference of the longitudinal change between Ins2+/+ and Ins2+/− and top-ranked correlation with BS in plasma amino acids. The experimental time-courses were obtained for Ins2+/+ and Ins2+/− as described above.

When calculating the time-course for each candidate parameter set and evaluating the average relative error from the experimental data, the measured time courses of the plasma amino acids were given for each experimental condition.

The calculation with the genetic algorithm was performed with a population of 300 in one generation, and one trial with 50,000 generations was repeated 50 times. From the last generation of each trial, the parameter set with the network structure consistent with the least average relative error between the calculated value and experimental value per sampling point was selected as the candidate network model, and from 50 candidates, one parameter set with the least average error was selected as the final candidate network model. This final candidate was subjected to the next sensitivity analysis.

### Sensitivity analysis method

To analyze the importance of each interrelated path in the estimated network, we evaluated the sensitivity of each parameter in the network. If the sensitivity of a certain parameter is high, then the corresponding path must be essential or rigid for realizing the experimentally observed time courses.

We added a 5% perturbation to each interrelated coefficient, *g*_*ij*_ or *h*_*ij*_, in the network and the relative squared error between the calculated time course data before adding perturbations, and the result after adding the perturbations was evaluated. Here, we defined the evaluated value of the sensitivity *S*_*gij*_ (*S*_*hij*_) for the interaction coefficient *g*_*ij*_ (*h*_*ij*_) as follows:3

where CAL_*d,i,t*_ shows the numerically calculated time course before adding a 5% perturbation at time *t* of the state variable *X*_*i*_ in the *d-th* data set, and PER_*d,i,t*_ shows the results after adding a 5% perturbation at time *t* of the state variable *X*_*i*_ in the *d-th* data set. *D* is the amount of the time course data, *N* is the number of variables, and *T* is the number of sampling points in each time course. For convenience of comparison, we performed scaling such that the highest evaluated value on the network candidate was 100, and other scores are expressed as the ratio to the highest value.

## Abbreviations

ABA: Alpha-aminobutyric acid
Ala: Alanine
Arg: Arginine
Asn: Asparagine
Asp: Asparagine
BS: Blood sugar
Cit: Citrulline
Gln: Glutamine
Glu: Glutamate
Gly: Glycine
His: Histidine
Ile: Isoleucine
Leu: Leucine
Lys: Lysine
Met: Methionine
Orn: Ornithine
Phe: Phenylalanine
Pro: Proline
Ser: Serine
Tau: Taurine
Thr: Threonine
Trp: Tryptophan
Tyr: Tyrosine
Val: Valine.
